# The vibration study of a sandwich conical shell with a saturated FGP core

**DOI:** 10.1038/s41598-022-09043-w

**Published:** 2022-03-23

**Authors:** Mohsen Nasr Esfahani, Mohammad Hashemian, Farshid Aghadavoudi

**Affiliations:** Department of Mechanical Engineering, Khomeinishahr Branch, Islamic Azad University, Khomeinishahr, 84175-119 Isfahan, Iran

**Keywords:** Engineering, Nanoscience and technology, Physics

## Abstract

This paper is provided to analyze the free vibration of a sandwich truncated conical shell with a saturated functionally graded porous (FGP) core and two same homogenous isotropic face sheets. The mechanical behavior of the saturated FGP is assumed based on Biot’s theory, the shell is modeled via the first-order shear deformation theory (FSDT), and the governing equations and boundary conditions are derived utilizing Hamilton’s principle. Three different porosity distribution patterns are studied including one homogenous uniform distribution pattern and two non-homogenous symmetric ones. The porosity parameters in mentioned distribution patterns are regulated to make them the same in the shell’s mass. The equations of motion are solved exactly in the circumferential direction via proper sinusoidal and cosinusoidal functions, and a numerical solution is provided in the meridional direction utilizing the differential quadrature method (DQM). The precision of the model is approved and the influences of several parameters such as circumferential wave number, the thickness of the FGP core, porosity parameter, porosity distribution pattern, the compressibility of the pore fluid, and boundary conditions on the shell’s natural frequencies are investigated. It is shown that the highest natural frequencies usually can be achieved when the larger pores are located close to the shell’s middle surface and in each vibrational mode, there is a special value of the porosity parameter which leads to the lowest natural frequencies. It is deduced that in most cases, natural frequencies decrease by increasing the thickness of the FGP core. In addition, reducing the compressibility of the porefluid a small growth in the natural frequencies can be seen.

## Introduction

Due to the numerous use of the conical shells in different engineering applications such as aerospace and mechanical engineering, high-power aircraft jet engines, high-speed centrifugal separators, and gas turbines, a considerable number of investigations have been presented on the mechanical analysis of such structures, recently. Sofiyev^1^ investigated the stability and free vibration analyses of heterogenous composite truncated conical shells reinforced with carbon nanotubes (CNTs) subjected to an axial load. He examined the effects of the percentage of the CNTs and heterogeneity on the buckling and free vibrational characteristics of the shell. The free vibration characteristics of rotating polymeric truncated conical shells enriched by graphene nanoplatelets (GNPs) were investigated by Afshari^2^. It was shown by him that the sequence of vibrational modes can be affected by the variation of semi-vertex angle. By utilizing analytical and numerical techniques and experimental tests, the free vibration study of conical shells stiffened by bevel stiffeners was examined by Zarei et al.^3^. They studied the influences of the shell’s geometrical characteristics on the natural frequencies of such a structure. Yousefi et al.^4,5^ studied the forced and free vibrational behavior of three-phase CNT/polymer/fiber truncated conical panels and shells. It was revealed by them that the larger length and higher embracing and semi-vertex angles result in the smaller natural frequencies. To complete these works, they hired particle swarm optimization to find the best values of mass fractions of the CNTs and fibers and orientation of the fibers to minimize the cost and maximize the fundamental frequency of the three-phase CNT/polymer/fiber laminated truncated conical panels^6^. Aris and Ahmadi^7^ studied the analysis of the nonlinear resonance of FGM (functionally graded materials) truncated conical shells exposed to an external harmonic excitation and thermal loading. They examined the effects of the shell’s geometrical characteristics and temperature on the nonlinear vibrational characteristics of the shell. By incorporating the agglomeration of the CNTs, the free vibration study of a CNT-reinforced spinning truncated conical shell was examined by Afshari and Amirabadi^8^. It was shown by them that variation of the rotational speed may change the sequence of the vibrational modes. The vibration study of combined conical-ribbed cylindrical-conical shell structures was investigated by Zhang et al.^9^. They approved the precision of their work by comparing their results with the corresponding ones obtained via the finite element method (FEM) and experiment tests. Fares et al.^10^ employed a layerwise formulation and analyzed the free vibration of multilayered CNT-reinforced truncated conical shells. They checked the dependency of the natural frequencies on the thickness stretching strains. For various boundary conditions, the natural frequencies of porous metal foam truncated conical shells were reported by Li et al.^11^. They examined the effects of the porosity parameter and pore dispersion pattern on the shell’s natural frequencies. Using FEM, Singha et al.^12^ analyzed the free vibration of rotating pre-twisted sandwich conical shells with a homogenous core and FG graphene-reinforced face sheets in a thermal environment. They studied the effect of graphene distribution patterns on natural frequencies. Adab et al.^13,14^ investigated the free vibrational behavior of non-rotating and rotating sandwich truncated conical microshells with an FGP core and GNP-reinforced face sheets. It was shown by them that the highest natural frequencies can be achieved when the big pores are located close to the middle surface of the microshell. Nasution et al.^15^ succeeded to find a semi-analytical solution for the supersonic flutter behavior of three-phase polymer/GNP/fiber laminated joined conical-conical shells. They concluded that the aeroelastic stability and flutter mode of such structures can be easily affected by the semi-vertex angles and lengths of the shell segments.

Due to some superior properties such as low density, high capacity of energy loss, low thermal conductivity, and high recyclability, porous materials have been receiving great interest as engineering materials in the transportation industry and mechanical, civil, and aerospace engineering^16^. The porous materials consist of two phases: the main phase is solid and the other one is either gas or liquid which can be found in nature such as layers of dust, stone, and wood^17^. The initial research on the mechanical characteristics of porous materials was performed by Biot^18–21^ when he proposed the constitutive relations in porous mediums known as Biot’s theory. Some authors studied the mechanical analysis of the beams, plates, shells, and panels made of porous materials or the sandwich structures with an FGP core. Leclaire et al.^22^ analyzed the dynamics of porous plates and studied the influences of porosity parameter and compressibility of pore fluid on the natural frequencies of such a plate. Kiani^23^ provided the dynamic response of porous beams subjected to the action of a moving load. The influences of the speed of moving load, length to thickness ratio of the beam, and compressibility of pore fluid on the vibration amplitude were examined by him. Xiang et al.^24^ investigated the forced vibrational behavior of thin rectangular porous plates. For various types of excitation, two selected boundary conditions, and two selected pore distribution patterns, they provided the vibration amplitude response versus excitation frequency. The mechanical buckling, static bending, and free vibration analyses of FG-porous beams were studied by Fouda et al.^25^. They proposed two models to calculate the mechanical properties of the porous beam including an implicit model and an explicit one and investigated the dependencies of the mechanical characteristics (static deflection, natural frequencies, and critical buckling load) on the porosity parameter. Mojahedin et al.^26^ studied the thermoelastic response of porous beams. They studied the dependency of the thermoelastic characteristics of such beams on the pore fluid. An exact solution was presented by Nikkhoo et al.^27^ for the dynamic response of flexo-poro-elastic structures subjected to moving loads. The influences of the porosity parameter, viscosity of the pore fluid, the velocity of the moving loads, and stiffness of the foundation on the dynamic response of the beam were examined by them. Enayat et al.^28,29^ studied the mechanical buckling, static bending, and free vibration characteristics of porous nanobeams subjected to thermal loading. It was shown by them that by increasing the porosity parameter, the static deflection increases and the critical buckling load decrease; but the effect of the porosity parameter on the natural frequencies significantly depends on the pore distribution pattern. The free vibration and aeroelastic stability behaviors of sandwich cylindrical thick panels with saturated FGP core were examined by Akbari et al.^30,31^. It was shown by them that the higher porosity parameter results in the weaker aeroelastic stability of the panel. The free vibration and aeroelastic stability behaviors of GNP-reinforced porous cylindrical panels were investigated by Zhou et al.^32^. They deduced that the critical speed of the panel diminishes by increasing the porosity parameter. Due to the weak effect of the pore fluid, many authors studied the mechanical analysis of the FGP structures by ignoring the pore fluid. For example, Chen et al.^33,34^ focused on the static bending and mechanical buckling behavior and also the free and forced vibration characteristics of FGP beams. They studied the effect of the porosity parameter on the static and dynamic deflection, critical buckling load, and the natural frequencies of the FGP beams. In another paper, they studied the static bending and mechanical buckling analyses of a novel FGP plate^35^. They proposed this novel type of porous material to achieve a smooth stress distribution through the plate thickness.

As stated, conical shells have been used in many fields regarding mechanical and aerospace applications such as high-speed centrifugal separators, gas turbines, and high-power aircraft jet engines. Thus, a reduction in the total weight of such structures is an interesting engineering problem. This goal is achieved in this paper using porous materials. The above literature review confirms that dynamic characteristics of the conical shells made of various types of materials have been investigated in the previous works, but the effect of the fluid-filled porous materials as the core in a sandwich truncated conical shell is not investigated. To the author’s best knowledge, the presented paper is the first attempt to analyze the free vibration of sandwich truncated conical shells with two isotropic homogenous face sheets and a saturated FGP core. The dependencies of the natural frequencies on the circumferential wave number, boundary conditions, compressibility of the pore fluid, porosity distribution pattern, porosity parameter, and the thickness of the FGP core are examined.

## Mathematical modeling

### Geometrical description

**Figure 1:**
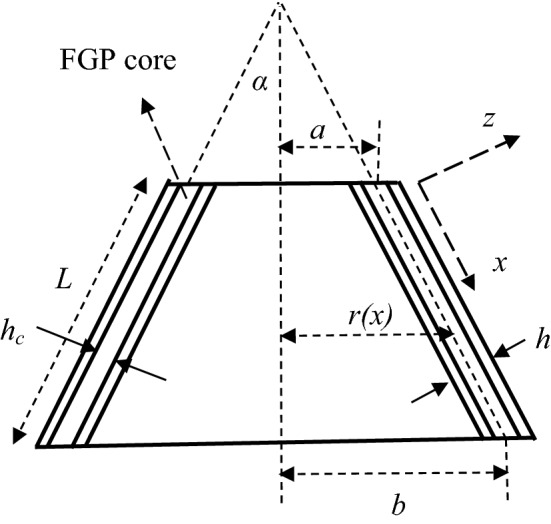
A sandwich truncated conical shell with an FGP core.

As Fig. 1 shows, a sandwich truncated conical shell is considered of thickness *h,* small radius *a,* length *L,* semi-vertex angle *α*, and large radius *b* = *a* + *L*sin*α*. The shell consists of a saturated FGP core of thickness *h*_c_ and two same homogenous isotropic face sheets of thickness *h*_f_ = 0.5(*h* − *h*_c_).

### Material properties

**Figure 2:**
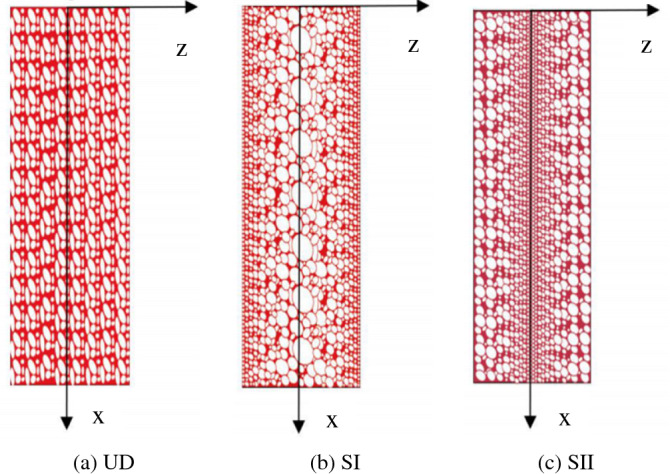
Porosity distribution patterns^37^.

As depicted in Fig. 2, three different porosity distribution patterns are considered in this paper for the shell’s core including a uniform porosity distribution (UD) and two non-uniform ones (SI and SII). The elastic modulus of the FGP core varies along thickness direction as^36^

1 $$\begin{array}{*{20}l} {UD:} & {E_{c} = \left( {1 - e_{0} } \right)E_{0} ,} \\ {SI:} & {E_{c} \left( z \right) = \left[ {1 - e_{1} \cos \left( {\frac{\pi z}{{h_{c} }}} \right)} \right]E_{0} ,} \\ {SII} & {E_{c} \left( z \right) = \left\{ {1 - e_{2} \left[ {1 - \cos \left( {\frac{\pi z}{{h_{c} }}} \right)} \right]} \right\}E_{0} ,} \\ \end{array}$$

where $E_{0}$ is the modulus of elasticity of the material with no porosity and *e*_0_, *e*_1_, and *e*_2_ stand for the porosity parameters.

To have a fair comparison between these porosity distribution patterns, it is better to regulate the porosity parameters to create the same value of mass. Utilizing the following equation between density (*ρ*) and the elastic modulus^37^

2 $$\frac{{E_{c} \left( z \right)}}{{E_{0} }} = \left[ {\frac{{\rho_{c} \left( z \right)}}{{\rho_{0} }}} \right]^{2.73} ,$$

in which *ρ*_*0*_ represents the density of the material with no porosity, one can write

3 $$\begin{array}{*{20}l} {\int\limits_{0}^{{\frac{{h_{c} }}{2}}} {\left( {1 - e_{0} } \right)^{{\frac{1}{2.73}}} dz} = \int\limits_{0}^{{\frac{{h_{c} }}{2}}} {\left[ {1 - e_{1} \cos \left( {\frac{\pi z}{{h_{c} }}} \right)} \right]^{{\frac{1}{2.73}}} dz} ,} \hfill \\ {\int\limits_{0}^{{\frac{{h_{c} }}{2}}} {\left\{ {1 - e_{2} \left[ {1 - \cos \left( {\frac{\pi z}{{h_{c} }}} \right)} \right]} \right\}^{{\frac{1}{2.73}}} dz} = \int\limits_{0}^{{\frac{{h_{c} }}{2}}} {\left[ {1 - e_{1} \cos \left( {\frac{\pi z}{{h_{c} }}} \right)} \right]^{{\frac{1}{2.73}}} dz} .} \hfill \\ \end{array}$$

For some values of the porosity parameter *e*_1_, the corresponding values of the porosity parameters *e*_*0*_ and *e*_*2*_ can be found in Table 1 and can be approximately stated as bellow^36^:

4 $$\begin{array}{*{20}l} e_{0} = 1.944e_{1}^{6} - 3.417e_{1}^{5} + 2.278e_{1}^{4} - 0.6708e_{1}^{3} + 0.122e_{1}^{2} + 0.6362e_{1} , \hfill \\ e_{2} = - \;0.4269e_{1}^{3} - 0.009286e_{1}^{2} + 1.732e_{1} . \hfill \\ \end{array}$$

**Table 1:** Regulating the porosity parameters for various distributions patterns^37^.

*e*_1_	0	0.1	0.2	0.3	0.4	0.5	0.6
*e*_0_	0	0.0640	0.1287	0.1942	0.2609	0.3289	0.3988
*e*_2_	0	0.1734	0.3426	0.5065	0.6637	0.8112	0.9432

Based on Biot’s poroelasticity theory for isotropic poroelastic mediums, the constitutive relations are stated as^16^

5 $$\begin{array}{*{20}l} {\sigma_{ij} = 2G\varepsilon_{ij} + \lambda_{u} \varepsilon_{kk} \delta_{ij} - \alpha_{0} p\delta_{ij} ,} & {i,j = 1,2,3,} \\ \end{array}$$

in which *σ*_ij_ and *ε*_ij_ sequentially stand for the stress and strain tensors and *α*_*0*_ is the Biot coefficient of effective stress. Also, with the following definitions, *G*, *λ*_u_, *p*, *ε*_kk_, and *δ*_ij_ represent the shear modulus, undrained Lame parameter, pore fluid pressure, volumetric strain, and the Kronecker delta:

6 $$\begin{array}{*{20}l} {G = \frac{E}{{2\left( {1 + \nu } \right)}},} & {\lambda_{u} = \frac{{2\nu_{u} }}{{1 - 2\nu_{u} }}G,} & {p = M\left( {\xi - \alpha_{0} \varepsilon_{kk} } \right),} & {\varepsilon_{kk} = \varepsilon_{xx} + \varepsilon_{\theta \theta } + \varepsilon_{zz} ,} & {\delta_{ij} = \left\{ {\begin{array}{*{20}l} 1 & {i = j} \\ 0 & {i \ne j} \\ \end{array} } \right.,} \\ \end{array}$$

in which *ξ* stands for the variation of fluid volume content and *ν*_u_ and *M* sequentially represent undrained Poisson’s ratio and Biot’s modulus and are defined as below:

7 $$\begin{array}{*{20}l} {\nu_{u} = \frac{{3\nu + \alpha_{0} B_{0} \left( {1 - 2\nu } \right)}}{{3 - \alpha_{0} B_{0} \left( {1 - 2\nu } \right)}},} & {M = \frac{{2\left( {\nu_{u} - \nu } \right)}}{{\alpha_{0}^{2} \left( {1 - 2\nu } \right)\left( {1 - 2\nu_{u} } \right)}}G,} \\ \end{array}$$

where *B*_0_ is known as the Skempton coefficient which deputizes the compressibility of the pore fluid. By increasing the Skempton coefficient from zero to one, the pore fluid varies from a completely compressible fluid to a noncompressible fluid.

For the undrained condition (*ξ* = 0), the plane stress assumption (*σ*_zz_ = 0), and employing Eqs. (6) and (7), Eq. (5) can be stated in *x*-*θ*-*z* coordinates as below (*γ*_ij_ = 2*ε*_ij_):

8 $$\left\{ {\begin{array}{*{20}l} {\sigma_{xx} } \\ {\sigma_{\theta \theta } } \\ {\sigma_{\theta z} } \\ {\sigma_{xz} } \\ {\sigma_{x\theta } } \\ \end{array} } \right\} = \left[ {\begin{array}{*{20}l} {C_{11} } & {C_{12} } & 0 & 0 & 0 \\ {C_{12} } & {C_{22} } & 0 & 0 & 0 \\ 0 & 0 & {k_{s} C_{44} } & 0 & 0 \\ 0 & 0 & 0 & {k_{s} C_{55} } & 0 \\ 0 & 0 & 0 & 0 & {C_{66} } \\ \end{array} } \right]\left\{ {\begin{array}{*{20}l} {\varepsilon_{xx} } \\ {\varepsilon_{\theta \theta } } \\ {\gamma_{\theta z} } \\ {\gamma_{xz} } \\ {\gamma_{x\theta } } \\ \end{array} } \right\},$$

where *k*_*s*_ = 5/6 is known as the shear correction factor and

9 $$\begin{array}{*{20}l} {\begin{array}{*{20}l} {C_{11} = C_{22} = 2f_{1} G,} & {C_{12} = 2f_{2} G,} & {C_{44} = C_{55} = C_{66} = G,} \\ \end{array} } \\ {\begin{array}{*{20}l} {f_{1} = \frac{{1 - 4\nu + 2\nu_{u} }}{{1 - 3\nu + 2\nu \nu_{u} }},} & {f_{2} = \frac{{2\left( {1 - \nu } \right)\nu_{u} - \nu }}{{1 - 3\nu + 2\nu \nu_{u} }}.} \\ \end{array} } \\ \end{array}$$

For the face sheets (*e*_1_ = *e*_2_ = *e*_3_ = 0 and *α*_0_ = 0, and as a result *ν*_u_ = *ν*), Eq. (9) can be stated as below:

10 $$\begin{array}{*{20}l} {C_{11} = C_{22} = \frac{{E_{f} }}{{1 - \nu_{f}^{2} }},} & {C_{12} = \nu_{f} \,C_{11} ,} & {C_{44} = C_{55} = C_{66} = G_{f} = \frac{{E_{f} }}{{2\left( {1 + \nu_{f} } \right)}},} \\ \end{array}$$

where the subscript *f* represents the mechanical properties of the isotropic homogenous face sheets.

### Governing equations

As stated in the FSDT, the following relation can be utilized for the displacement field^38^:

11 $$\begin{array}{*{20}l} u_{1} \left( {x,\theta ,z,t} \right) = z\varphi_{x} \left( {x,\theta ,t} \right) + u\left( {x,\theta ,t} \right), \hfill \\ u_{2} \left( {x,\theta ,z,t} \right) = z\varphi_{\theta } \left( {x,\theta ,t} \right) + v\left( {x,\theta ,t} \right), \hfill \\ u_{3} \left( {x,\theta ,z,t} \right) = w\left( {x,\theta ,t} \right). \hfill \\ \end{array}$$

in which *u*_1_, *u*_2_*,* and *u*_3_ sequentially are the displacement components along of *x*, *θ*, and *z*, directions; *u*, *v*, and *w* show the corresponding components of displacement at the middle surface (*z* = 0); and *φ*_*x*_ and *φ*_*θ*_ stand for the rotations about *θ*- and *x*-axes, sequentially.

The non-zero components of the strain tensor (ε_ij_) can be presented as below^38^:

12 $$\begin{array}{*{20}l} {\varepsilon_{xx} = \frac{\partial u}{{\partial x}} + z\frac{{\partial \varphi_{x} }}{\partial x},} {\varepsilon_{\theta \theta } = \frac{\sin \alpha }{r}u + \frac{\cos \alpha }{r}w + \frac{1}{r}\frac{\partial v}{{\partial \theta }} + z\left( {\frac{\sin \alpha }{r}\varphi_{x} + \frac{1}{r}\frac{{\partial \varphi_{\theta } }}{\partial \theta }} \right),} \\ \hfill \\ {\gamma_{\theta z} = - \frac{\cos \alpha }{r}v + \frac{1}{r}\frac{\partial w}{{\partial \theta }} + \varphi_{\theta } ,} {\gamma_{xz} = \frac{\partial w}{{\partial x}} + \varphi_{x} ,} \\ \hfill \\ \gamma_{x\theta } = \frac{1}{r}\frac{\partial u}{{\partial \theta }} - \frac{\sin \alpha }{r}v + \frac{\partial v}{{\partial x}} + z\left( {\frac{1}{r}\frac{{\partial \varphi_{x} }}{\partial \theta } - \frac{\sin \alpha }{r}\varphi_{\theta } + \frac{{\partial \varphi_{\theta } }}{\partial x}} \right). \hfill \\ \end{array}$$

The governing equations and boundary conditions can be derived utilizing Hamilton’s principle as below^39^:

13 $$\int\limits_{{t_{1} }}^{{t_{2} }} {\left( {\delta W_{n.c.} + \delta T - \delta U_{s} } \right)dt} = 0,$$

in which [*t*_1_,*t*_2_] is an arbitrary time interval, *δ* shows the variational operator, *T* shows kinetic energy, *U*_*s*_ is strain energy, and *W*_*n.c.*_ stands for the work done by external non-conservative loads.

The shell’s kinetic energy can be presented as below:

14 $$T = \frac{1}{2}\iiint\limits_{V} {\rho \left[ {\left( {\frac{{\partial u_{1} }}{\partial t}} \right)^{2} + \left( {\frac{{\partial u_{2} }}{\partial t}} \right)^{2} + \left( {\frac{{\partial u_{3} }}{\partial t}} \right)^{2} } \right]dV},$$

where

15 $$\iiint\limits_{V} {\left( {} \right)dV} = \iint\limits_{S} {\int\limits_{{ - \frac{h}{2}}}^{\frac{h}{2}} {\left( {} \right)dzdS} },$$

in which d*S* = *r*d*x*d*θ* shows the shell’s surface.

Equation (14) can be represented using Eqs. (11) and (15) as below:

16 $$T = \frac{1}{2}\iint\limits_{S} {\left\{ {I_{0} \left[ {\left( {\frac{\partial u}{{\partial t}}} \right)^{2} + \left( {\frac{\partial v}{{\partial t}}} \right)^{2} + \left( {\frac{\partial w}{{\partial t}}} \right)^{2} } \right] + 2I_{1} \left( {\frac{\partial u}{{\partial t}}\frac{{\partial \varphi_{x} }}{\partial t} + \frac{\partial v}{{\partial t}}\frac{{\partial \varphi_{\theta } }}{\partial t}} \right) + I_{2} \left[ {\left( {\frac{{\partial \varphi_{x} }}{\partial t}} \right)^{2} + \left( {\frac{{\partial \varphi_{\theta } }}{\partial t}} \right)^{2} } \right]} \right\}}dS,$$

where

17 $$\begin{array}{*{20}l} {I_{i} = \int\limits_{{ - \frac{h}{2}}}^{\frac{h}{2}} {\rho \left( z \right)z^{i} } dz,} & {i = 0,1,2.} \\ \end{array}$$

Due to the symmetry in pore distribution patterns, it is evident that *I*_1_ = 0, consequently, the variation of the shell’s kinetic energy can be stated as below:

18 $$\delta T = \iint\limits_{S} {\left[ {I_{0} \left( {\frac{\partial u}{{\partial t}}\frac{\partial \delta u}{{\partial t}} + \frac{\partial v}{{\partial t}}\frac{\partial \delta v}{{\partial t}} + \frac{\partial w}{{\partial t}}\frac{\partial \delta w}{{\partial t}}} \right) + I_{2} \left( {\frac{{\partial \varphi_{x} }}{\partial t}\frac{{\partial \delta \varphi_{x} }}{\partial t} + \frac{{\partial \varphi_{\theta } }}{\partial t}\frac{{\partial \delta \varphi_{\theta } }}{\partial t}} \right)} \right]}\,dS.$$

The variation of the shell’s strain energy can be calculated as below

19 $$\delta U_{s} = \iiint\limits_{V} {\sigma_{ij} \delta \varepsilon_{ij} dV},$$

which can be represented using Eqs. (12) and (15) as

20 $$\begin{gathered} \delta U_{s} = \iint\limits_{S} {\left( {N_{xx} \frac{\partial \delta u}{{\partial x}} + \frac{{N_{\theta \theta } }}{r}\frac{\partial \delta v}{{\partial \theta }} + \frac{{N_{\theta \theta } \sin \alpha }}{r}\delta u + \frac{{N_{\theta \theta } \cos \alpha }}{r}\delta w + M_{xx} \frac{{\partial \delta \varphi_{x} }}{\partial x} + \frac{{M_{\theta \theta } \sin \alpha }}{r}\delta \varphi_{x} } \right.} \hfill \\ + \frac{{M_{\theta \theta } }}{r}\frac{{\partial \delta \varphi_{\theta } }}{\partial \theta } - \frac{{Q_{\theta z} \cos \alpha }}{r}\delta v + \frac{{Q_{\theta z} }}{r}\frac{\partial \delta w}{{\partial \theta }} + Q_{\theta z} \delta \varphi_{\theta } + Q_{xz} \frac{\partial \delta w}{{\partial x}} + Q_{xz} \delta \varphi_{x} + \frac{{N_{x\theta } }}{r}\frac{\partial \delta u}{{\partial \theta }} \hfill \\ \left. { + N_{x\theta } \frac{\partial \delta v}{{\partial x}} - \frac{{N_{x\theta } \sin \alpha }}{r}\delta v + \frac{{M_{x\theta } }}{r}\frac{{\partial \delta \varphi_{x} }}{\partial \theta } + M_{x\theta } \frac{{\partial \delta \varphi_{\theta } }}{\partial x} - \frac{{M_{x\theta } \sin \alpha }}{r}\delta \varphi_{\theta } } \right)dS, \hfill \\ \end{gathered}$$

where

21 $$\begin{array}{*{20}l} {\left\{ {\begin{array}{*{20}l} {N_{xx} } \\ {N_{\theta \theta } } \\ {N_{x\theta } } \\ \end{array} } \right\} = \int\limits_{{ - \frac{h}{2}}}^{\frac{h}{2}} {\left\{ {\begin{array}{*{20}l} {\sigma_{xx} } \\ {\sigma_{\theta \theta } } \\ {\sigma_{x\theta } } \\ \end{array} } \right\}dz} ,} & {\left\{ {\begin{array}{*{20}l} {M_{xx} } \\ {M_{\theta \theta } } \\ {M_{x\theta } } \\ \end{array} } \right\} = \int\limits_{{ - \frac{h}{2}}}^{\frac{h}{2}} {\left\{ {\begin{array}{*{20}l} {\sigma_{xx} } \\ {\sigma_{\theta \theta } } \\ {\sigma_{x\theta } } \\ \end{array} } \right\}zdz} ,} & {\left\{ {\begin{array}{*{20}l} {Q_{xz} } \\ {Q_{\theta z} } \\ \end{array} } \right\} = \int\limits_{{ - \frac{h}{2}}}^{\frac{h}{2}} {\left\{ {\begin{array}{*{20}l} {\sigma_{xz} } \\ {\sigma_{\theta z} } \\ \end{array} } \right\}dz} .} \\ \end{array}$$

By substituting Eqs. (8) and (12) into Eq. (21), the following equation can be achieved:

22 $$\begin{array}{*{20}l} N_{xx} = A_{11} \frac{\partial u}{{\partial x}} + \frac{{A_{12} \sin \alpha }}{r}u + \frac{{A_{12} }}{r}\frac{\partial v}{{\partial \theta }} + \frac{{A_{12} \cos \alpha }}{r}w + B_{11} \frac{{\partial \varphi_{x} }}{\partial x} + \frac{{B_{12} \sin \alpha }}{r}\varphi_{x} + \frac{{B_{12} }}{r}\frac{{\partial \varphi_{\theta } }}{\partial \theta }, \hfill \\ N_{\theta \theta } = A_{12} \frac{\partial u}{{\partial x}} + \frac{{A_{22} \sin \alpha }}{r}u + \frac{{A_{22} }}{r}\frac{\partial v}{{\partial \theta }} + \frac{{A_{22} \cos \alpha }}{r}w + B_{12} \frac{{\partial \varphi_{x} }}{\partial x} + \frac{{B_{22} \sin \alpha }}{r}\varphi_{x} + \frac{{B_{22} }}{r}\frac{{\partial \varphi_{\theta } }}{\partial \theta }, \hfill \\ M_{xx} = B_{11} \frac{\partial u}{{\partial x}} + \frac{{B_{12} \sin \alpha }}{r}u + \frac{{B_{12} }}{r}\frac{\partial v}{{\partial \theta }} + \frac{{B_{12} \cos \alpha }}{r}w + D_{11} \frac{{\partial \varphi_{x} }}{\partial x} + \frac{{D_{12} \sin \alpha }}{r}\varphi_{x} + \frac{{D_{12} }}{r}\frac{{\partial \varphi_{\theta } }}{\partial \theta }, \hfill \\ M_{\theta \theta } = B_{12} \frac{\partial u}{{\partial x}} + \frac{{B_{22} \sin \alpha }}{r}u + \frac{{B_{22} }}{r}\frac{\partial v}{{\partial \theta }} + \frac{{B_{22} \cos \alpha }}{r}w + D_{12} \frac{{\partial \varphi_{x} }}{\partial x} + \frac{{D_{22} \sin \alpha }}{r}\varphi_{x} + \frac{{D_{22} }}{r}\frac{{\partial \varphi_{\theta } }}{\partial \theta }, \hfill \\ N_{x\theta } = \frac{{A_{66} }}{r}\frac{\partial u}{{\partial \theta }} + A_{66} \frac{\partial v}{{\partial x}} - \frac{{A_{66} \sin \alpha }}{r}v + \frac{{B_{66} }}{r}\frac{{\partial \varphi_{x} }}{\partial \theta } + B_{66} \frac{{\partial \varphi_{\theta } }}{\partial x} - \frac{{B_{66} \sin \alpha }}{r}\varphi_{\theta } , \hfill \\ M_{x\theta } = \frac{{B_{66} }}{r}\frac{\partial u}{{\partial \theta }} + B_{66} \frac{\partial v}{{\partial x}} - \frac{{B_{66} \sin \alpha }}{r}v + \frac{{D_{66} }}{r}\frac{{\partial \varphi_{x} }}{\partial \theta } + D_{66} \frac{{\partial \varphi_{\theta } }}{\partial x} - \frac{{D_{66} \sin \alpha }}{r}\varphi_{\theta } , \hfill \\ Q_{\theta z} = A_{44} \left( { - \frac{\cos \alpha }{r}v + \frac{1}{r}\frac{\partial w}{{\partial \theta }} + \varphi_{\theta } } \right), \hfill \\ Q_{xz} = A_{55} \left( {\frac{\partial w}{{\partial x}} + \varphi_{x} } \right), \hfill \\ \end{array}$$

where (i,j = 1, 2, 6)

23 $$\begin{array}{*{20}l} {\left\{ {\begin{array}{*{20}l} {A_{ij} } \\ {B_{ij} } \\ {D_{ij} } \\ \end{array} } \right\} = \int\limits_{{ - \frac{h}{2}}}^{\frac{h}{2}} {C_{ij} \left( z \right)\left\{ {\begin{array}{*{20}l} 1 \\ z \\ {z^{2} } \\ \end{array} } \right\}dz} ,} & {\left\{ {\begin{array}{*{20}l} {A_{44} } \\ {A_{55} } \\ \end{array} } \right\} = k_{s} \int\limits_{{ - \frac{h}{2}}}^{\frac{h}{2}} {\left\{ {\begin{array}{*{20}l} {C_{44} \left( z \right)} \\ {C_{55} \left( z \right)} \\ \end{array} } \right\}dz} .} \\ \end{array}$$

Due to the symmetry in pore distribution patterns, it is evident that *B*_ij_ = 0, consequently, Eq. (22) can be simplified as follows:

24 $$\begin{array}{*{20}l} {N_{xx} = A_{11} \frac{\partial u}{{\partial x}} + \frac{{A_{12} \sin \alpha }}{r}u + \frac{{A_{12} }}{r}\frac{\partial v}{{\partial \theta }} + \frac{{A_{12} \cos \alpha }}{r}w,} {M_{xx} = D_{11} \frac{{\partial \varphi_{x} }}{\partial x} + \frac{{D_{12} \sin \alpha }}{r}\varphi_{x} + \frac{{D_{12} }}{r}\frac{{\partial \varphi_{\theta } }}{\partial \theta },} \\ \hfill \\ {N_{\theta \theta } = A_{12} \frac{\partial u}{{\partial x}} + \frac{{A_{22} \sin \alpha }}{r}u + \frac{{A_{22} }}{r}\frac{\partial v}{{\partial \theta }} + \frac{{A_{22} \cos \alpha }}{r}w,} {M_{\theta \theta } = D_{12} \frac{{\partial \varphi_{x} }}{\partial x} + \frac{{D_{22} \sin \alpha }}{r}\varphi_{x} + \frac{{D_{22} }}{r}\frac{{\partial \varphi_{\theta } }}{\partial \theta },} \\ \hfill \\ {N_{x\theta } = \frac{{A_{66} }}{r}\frac{\partial u}{{\partial \theta }} + A_{66} \frac{\partial v}{{\partial x}} - \frac{{A_{66} \sin \alpha }}{r}v,} {M_{x\theta } = \frac{{D_{66} }}{r}\frac{{\partial \varphi_{x} }}{\partial \theta } + D_{66} \frac{{\partial \varphi_{\theta } }}{\partial x} - \frac{{D_{66} \sin \alpha }}{r}\varphi_{\theta } ,} \\ \hfill \\ {Q_{\theta z} = A_{44} \left( { - \frac{\cos \alpha }{r}v + \frac{1}{r}\frac{\partial w}{{\partial \theta }} + \varphi_{\theta } } \right),} {Q_{xz} = A_{55} \left( {\frac{\partial w}{{\partial x}} + \varphi_{x} } \right).} \\ \hfill \\ \end{array}$$

In the free vibration analysis, the shell is not subjected to any external load (*δW*_*n.c.*_ = 0); consequently, by substituting Eqs. (18) and (20) into Eq. (13), the following set of the governing equations can be achieved:

25 $$\begin{array}{*{20}l} \frac{{\partial N_{xx} }}{\partial x} + \frac{\sin \alpha }{r}\left( {N_{xx} - N_{\theta \theta } } \right) + \frac{1}{r}\frac{{\partial N_{x\theta } }}{\partial \theta } - I_{0} \frac{{\partial^{2} u}}{{\partial t^{2} }} = 0, \hfill \\ \frac{1}{r}\frac{{\partial N_{\theta \theta } }}{\partial \theta } + \frac{{\partial N_{x\theta } }}{\partial x} + \frac{2\sin \alpha }{r}N_{x\theta } + \frac{\cos \alpha }{r}Q_{\theta z} - I_{0} \frac{{\partial^{2} v}}{{\partial t^{2} }} = 0, \hfill \\ \frac{{\partial Q_{xz} }}{\partial x} + \frac{\sin \alpha }{r}Q_{xz} + \frac{1}{r}\frac{{\partial Q_{\theta z} }}{\partial \theta } - \frac{\cos \alpha }{r}N_{\theta \theta } - I_{0} \frac{{\partial^{2} w}}{{\partial t^{2} }} = 0, \hfill \\ \frac{{\partial M_{xx} }}{\partial x} + \frac{\sin \alpha }{r}\left( {M_{xx} - M_{\theta \theta } } \right) + \frac{1}{r}\frac{{\partial M_{x\theta } }}{\partial \theta } - Q_{xz} - I_{2} \frac{{\partial^{2} \varphi_{x} }}{{\partial t^{2} }} = 0, \hfill \\ \frac{1}{r}\frac{{\partial M_{\theta \theta } }}{\partial \theta } + \frac{{\partial M_{x\theta } }}{\partial x} + \frac{2\sin \alpha }{r}M_{x\theta } - Q_{\theta z} - I_{2} \frac{{\partial^{2} \varphi_{\theta } }}{{\partial t^{2} }} = 0, \hfill \\ \end{array}$$

and the corresponding boundary condition can be stated as below:

26 $$\begin{array}{*{20}l} {{\text{Clamped}}\,\left( {\text{C}} \right){\text{:}}\;u = v = w = 0,\;\psi _{x} = \psi _{\theta } = 0,} \hfill \\ {{\text{Simply}}\,{\text{supported}}\,\left( {\text{S}} \right){\text{:}}\;N_{{xx}} = 0,\;v = w = 0,\;M_{{xx}} = 0,\;\psi _{\theta } = 0,} \hfill \\ {{\text{Free}}\,\left( {\text{F}} \right){\text{:}}\;N_{{xx}} = N_{{x\theta }} = Q_{{xz}} = 0,\;M_{{xx}} = M_{{x\theta }} = 0.} \hfill \\ \end{array}$$

By substituting Eq. (24) into Eq. (25) and employing the following solution:

27 $$\begin{array}{*{20}l} {\left\{ {\begin{array}{*{20}l} {u\left( {x,\theta ,t} \right)} \\ {v\left( {x,\theta ,t} \right)} \\ {w\left( {x,\theta ,t} \right)} \\ {\varphi_{x} \left( {x,\theta ,t} \right)} \\ {\varphi_{\theta } \left( {x,\theta ,t} \right)} \\ \end{array} } \right\} = \left\{ {\begin{array}{*{20}l} {U\left( x \right)e^{i\omega t} \cos \left( {n\theta } \right)} \\ {V\left( x \right)e^{i\omega t} \sin \left( {n\theta } \right)} \\ {W\left( x \right)e^{i\omega t} \cos \left( {n\theta } \right)} \\ {X\left( x \right)e^{i\omega t} \cos \left( {n\theta } \right)} \\ {\Theta \left( x \right)e^{i\omega t} \sin \left( {n\theta } \right)} \\ \end{array} } \right\},} & {n = 1,2,3, \ldots ,} \\ \end{array}$$

in which *ω* is an eigenvalue and *n* is called the circumferential wave number, the set of the governing equations can be represented as below:

28 $$\begin{aligned} A_{11} U^{\prime\prime} + & \frac{{A_{11} \sin \alpha }}{r}U^{\prime} - \frac{{A_{22} \sin^{2} \alpha + A_{66} n^{2} }}{{r^{2} }}U + \frac{{n\left( {A_{12} + A_{66} } \right)}}{r}V^{\prime} \\ - & \frac{{n\left( {A_{22} + A_{66} } \right)\sin \alpha }}{{r^{2} }}V + \frac{{A_{12} \cos \alpha }}{r}W^{\prime} - \frac{{A_{22} \sin 2\alpha }}{{2r^{2} }}W + I_{0} \omega^{2} U = 0, \\ - & \frac{{n\left( {A_{12} + A_{66} } \right)}}{r}U^{\prime} - \frac{{n\left( {A_{22} + A_{66} } \right)\sin \alpha }}{{r^{2} }}U + A_{66} V^{\prime\prime} + \frac{{A_{66} \sin \alpha }}{r}V^{\prime} \\ - & \frac{{A_{22} n^{2} + A_{44} \cos^{2} \alpha + A_{66} \sin^{2} \alpha }}{{r^{2} }}V - \frac{{n\left( {A_{22} + A_{44} } \right)\cos \alpha }}{{r^{2} }}W + \frac{{A_{44} \cos \alpha }}{r}\Theta + I_{0} \omega^{2} V = 0, \\ - & \frac{{A_{12} \cos \alpha }}{r}U^{\prime} - \frac{{A_{22} \sin 2\alpha }}{{2r^{2} }}U - \frac{{n\left( {A_{22} + A_{44} } \right)\cos \alpha }}{{r^{2} }}V + A_{55} W^{\prime\prime} + \frac{{A_{55} \sin \alpha }}{r}W^{\prime} \\ - & \frac{{A_{22} \cos^{2} \alpha + A_{44} n^{2} }}{{r^{2} }}W + A_{55} X^{\prime} + \frac{{A_{55} \sin \alpha }}{r}X + \frac{{nA_{44} }}{r}\Theta + I_{0} \omega^{2} W = 0, \\ - & A_{55} W^{\prime} + D_{11} X^{\prime\prime} + \frac{{D_{11} \sin \alpha }}{r}X^{\prime} - \left( {A_{55} + \frac{{D_{22} \sin^{2} \alpha + D_{66} n^{2} }}{{r^{2} }}} \right)X \\ + & \frac{{n\left( {D_{12} + D_{66} } \right)}}{r}\Theta^{\prime} - \frac{{n\left( {D_{22} + D_{66} } \right)\sin \alpha }}{{r^{2} }}\Theta + I_{2} \omega^{2} X = 0, \\ \frac{{A_{44} \cos \alpha }}{r}V + & \frac{{nA_{44} }}{r}W - \frac{{n\left( {D_{12} + D_{66} } \right)}}{r}X^{\prime} - \frac{{n\left( {D_{22} + D_{66} } \right)\sin \alpha }}{{r^{2} }}X + D_{66} \Theta^{\prime\prime} \\ + & \frac{{D_{66} \sin \alpha }}{r}\Theta^{\prime} - \left( {A_{44} + \frac{{D_{22} n^{2} + D_{66} \sin^{2} \alpha }}{{r^{2} }}} \right)\Theta + I_{2} \omega^{2} \Theta = 0, \\ \end{aligned}$$

where prime indicates derivative with respect to the spatial variable *x*.

Likewise, by substituting Eq. (24) and (27) into Eq. (26), the boundary conditions can be stated as below:

29 $$\begin{array}{*{20}l} {{\text{Clamped}}\,\left( {\text{C}} \right){\text{:}}\;U = V = W = 0,\;X = \Theta = 0,} \hfill \\ {{\text{Simply}}\,{\text{Supported}}\,\left( {\text{S}} \right){\text{:}}} \hfill \\ {A_{{11}} U^{\prime} + \frac{{A_{{12}} \sin \alpha }}{r}U = 0,\;V = W = 0,\;D_{{11}} X^{\prime} + \frac{{D_{{12}} \sin \alpha }}{r}X = 0,\;\Theta = 0,} \hfill \\ {Free\,\left( F \right):} \hfill \\ {A_{{11}} U^{\prime} + \frac{{A_{{12}} \sin \alpha }}{r}U + \frac{{nA_{{12}} }}{r}V + \frac{{A_{{12}} \cos \alpha }}{r}W = 0,\; - \frac{n}{r}U - \frac{{\sin \alpha }}{r}V + V^{\prime} = 0,} \hfill \\ {W^{\prime} + X = 0,\;D_{{11}} X^{\prime} + \frac{{D_{{12}} \sin \alpha }}{r}X + \frac{{nD_{{12}} }}{r}\Theta = 0,\; - \frac{n}{r}X + \Theta ^{\prime} - \frac{{\sin \alpha }}{r}\Theta = 0.} \hfill \\ \end{array}$$

## Solution methodology

In the current section, the DQM is employed as a well-accepted and well-known numerical approach to provide an approximate solution for the set of the governing Eqs. (28) with any combination of the boundary conditions (29) at both ends of the shell (*x* = 0,*L*). Based on the main idea in the DQM, each derivative of a function like *P*(*x*) can be estimated in terms of the weighted sum of its values at a set of grid points as below:

30 $$\left\{ {\frac{{d^{k} P}}{{dx^{k} }}} \right\} = \left[ {F^{\left( k \right)} } \right]\left\{ P \right\},$$

where [*F*^(k)^] is called the weighting coefficient matrix related to the *k*th order derivative and is presented as below^40^:

31 $$\begin{gathered} \begin{array}{*{20}l} {F_{ij}^{\left( 1 \right)} = \left\{ \begin{gathered} \frac{{\prod\limits_{\begin{subarray}{l} m = 1 \\ m \ne i,j \end{subarray} }^{N} {\left( {x_{i} - x_{m} } \right)} }}{{\prod\limits_{\begin{subarray}{l} m = 1 \\ m \ne j \end{subarray} }^{N} {\left( {x_{j} - x_{m} } \right)} }}\,\,\,,\,\,\,\,i \ne j \hfill \\ \sum\limits_{\begin{subarray}{l} m = 1 \\ m \ne i \end{subarray} }^{N} {\left( {x_{i} - x_{m} } \right)^{ - 1} \,\,,\,\,\,i = j} \hfill \\ \end{gathered} \right.} & {i,j = 1,2,...,N} \\ \end{array} , \hfill \\ \begin{array}{*{20}l} {\left[ {F^{\left( k \right)} } \right] = \left[ {F^{\left( 1 \right)} } \right]\left[ {F^{{\left( {k - 1} \right)}} } \right],} & {k = 2,3, \ldots \,.} \\ \end{array} \hfill \\ \end{gathered}$$

The distribution pattern of the grid points plays an important role in the convergence of the solution in the DQM. With the following definition for 0 ≤ *x* ≤ *L*, the Gauss–Lobatto–Chebyshev distribution pattern is utilized in this work^40^:

32 $$x_{i} = \frac{L}{2} - \frac{L}{2}\cos \left( {\frac{i - 1}{{N - 1}}\pi } \right).$$

By applying Eq. (30) and the following notation:

33 $$\begin{array}{*{20}l} {\left[ A \right] = \left[ {F^{\left( 1 \right)} } \right]} & {\left[ B \right] = \left[ {F^{\left( 2 \right)} } \right],} \\ \end{array}$$

the governing Eqs. (28) can be stated as below:

34 $$\left[ K \right]\left\{ c \right\} = \omega^{2} \left[ M \right]\left\{ c \right\},$$

where

35 $$\begin{array}{*{20}l} \begin{array}{*{20}l} {\left\{ c \right\}_{{5N \times 1}} = \left\{ {\begin{array}{*{20}l} {\left\{ U \right\}} \\ {\left\{ V \right\}} \\ {\left\{ W \right\}} \\ {\left\{ X \right\}} \\ {\left\{ \Theta \right\}} \\ \end{array} } \right\},} & {\left[ K \right] = \left[ {\begin{array}{*{20}l} {k_{{11}} } & {k_{{12}} } & {k_{{13}} } & {\left[ 0 \right]} & {\left[ 0 \right]} \\ {k_{{21}} } & {k_{{22}} } & {k_{{23}} } & {\left[ 0 \right]} & {k_{{25}} } \\ {k_{{31}} } & {k_{{32}} } & {k_{{33}} } & {k_{{34}} } & {k_{{35}} } \\ {\left[ 0 \right]} & {\left[ 0 \right]} & {k_{{43}} } & {k_{{44}} } & {k_{{45}} } \\ {\left[ 0 \right]} & {k_{{52}} } & {k_{{53}} } & {k_{{54}} } & {k_{{55}} } \\ \end{array} } \right],} & {\left[ M \right] = - \left[ {\begin{array}{*{20}l} {I_{0} I} & {\left[ 0 \right]} & {\left[ 0 \right]} & {\left[ 0 \right]} & {\left[ 0 \right]} \\ {\left[ 0 \right]} & {I_{0} I} & {\left[ 0 \right]} & {\left[ 0 \right]} & {\left[ 0 \right]} \\ {\left[ 0 \right]} & {\left[ 0 \right]} & {I_{0} I} & {\left[ 0 \right]} & {\left[ 0 \right]} \\ {\left[ 0 \right]} & {\left[ 0 \right]} & {\left[ 0 \right]} & {I_{2} I} & {\left[ 0 \right]} \\ {\left[ 0 \right]} & {\left[ 0 \right]} & {\left[ 0 \right]} & {\left[ 0 \right]} & {I_{2} I} \\ \end{array} } \right],} \\ \end{array} \hfill \\ k_{{11}} = A_{{11}} \left[ B \right] + A_{{11}} \sin \alpha \left[ {a_{1} } \right]\left[ A \right] - \left( {A_{{22}} \sin ^{2} \alpha + A_{{66}} n^{2} } \right)\left[ {a_{2} } \right]I, \hfill \\ k_{{12}} = n\left( {A_{{12}} + A_{{66}} } \right)\left[ {a_{1} } \right]\left[ A \right] - n\left( {A_{{22}} + A_{{66}} } \right)\sin \alpha \left[ {a_{2} } \right]I, \hfill \\ k_{{13}} = A_{{12}} \cos \alpha \left[ {a_{1} } \right]\left[ A \right] - 0.5A_{{22}} \sin 2\alpha \left[ {a_{2} } \right]I, \hfill \\ k_{{21}} = - n\left( {A_{{12}} + A_{{66}} } \right)\left[ {a_{1} } \right]\left[ A \right] - n\left( {A_{{22}} + A_{{66}} } \right)\sin \alpha \left[ {a_{2} } \right], \hfill \\ k_{{22}} = A_{{66}} \left[ B \right] + A_{{66}} \sin \alpha \left[ {a_{1} } \right]\left[ A \right] - \left( {A_{{22}} n^{2} + A_{{44}} \cos ^{2} \alpha + A_{{66}} \sin ^{2} \alpha } \right)\left[ {a_{2} } \right], \hfill \\ \begin{array}{*{20}l} {k_{{23}} = - n\left( {A_{{22}} + A_{{44}} } \right)\cos \alpha \left[ {a_{2} } \right],} & {k_{{25}} = A_{{44}} \cos \alpha \left[ {a_{1} } \right],} \\ \end{array} \hfill \\ \begin{array}{*{20}l} {k_{{31}} = - A_{{12}} \cos \alpha \left[ {a_{1} } \right]\left[ A \right] - 0.5A_{{22}} \sin 2\alpha \left[ {a_{2} } \right],} & {k_{{32}} = - n\left( {A_{{22}} + A_{{44}} } \right)\cos \alpha \left[ {a_{2} } \right],} \\ \end{array} \hfill \\ k_{{33}} = A_{{55}} \left[ B \right] + A_{{55}} \sin \alpha \left[ {a_{1} } \right]\left[ A \right] - \left( {A_{{22}} \cos ^{2} \alpha + A_{{44}} n^{2} } \right)\left[ {a_{2} } \right], \hfill \\ \begin{array}{*{20}l} {k_{{34}} = A_{{55}} \left[ A \right] + A_{{55}} \sin \alpha \left[ {a_{1} } \right],} & {k_{{35}} = nA_{{44}} \left[ {a_{1} } \right],} \\ \end{array} \hfill \\ \begin{array}{*{20}l} {k_{{43}} = - A_{{55}} \left[ A \right],} & {k_{{44}} = D_{{11}} \left[ B \right] + D_{{11}} \sin \alpha \left[ {a_{1} } \right]\left[ A \right] - A_{{55}} I - \left( {D_{{22}} \sin ^{2} \alpha + D_{{66}} n^{2} } \right)\left[ {a_{2} } \right],} \\ \end{array} \hfill \\ k_{{45}} = n\left( {D_{{12}} + D_{{66}} } \right)\left[ {a_{1} } \right]\left[ A \right] - n\left( {D_{{22}} + D_{{66}} } \right)\sin \alpha \left[ {a_{2} } \right],\begin{array}{*{20}l} {k_{{52}} = A_{{44}} \cos \alpha \left[ {a_{1} } \right],} & {k_{{53}} = nA_{{44}} \left[ {a_{1} } \right],} \\ \end{array} \hfill \\ k_{{54}} = - n\left( {D_{{12}} + D_{{66}} } \right)\left[ {a_{1} } \right]\left[ A \right] - n\left( {D_{{22}} + D_{{66}} } \right)\sin \alpha \left[ {a_{2} } \right], \hfill \\ k_{{55}} = D_{{66}} \left[ B \right] + D_{{66}} \sin \alpha \left[ {a_{1} } \right]\left[ A \right] - A_{{44}} I - \left( {D_{{22}} n^{2} + D_{{66}} \sin ^{2} \alpha } \right)\left[ {a_{2} } \right], \hfill \\ \end{array}$$

in which [a_1_] and [a_2_] are two diagonal matrices defined as below:

36 $$\begin{array}{*{20}l} {\left( {a_{1} } \right)_{ii} = \frac{1}{{r_{i} }},} & {\left( {a_{2} } \right)_{ii} = \frac{1}{{r_{i}^{2} }}.} \\ \end{array}$$

By applying Eqs. (30) and (32) on Eq. (29), the boundary conditions can be presented as below:

37 $$\left[ \Gamma \right]\left\{ c \right\} = \left\{ 0 \right\},$$

where

38 $$\left[ \Gamma \right]_{10 \times 5N} = \left[ {\begin{array}{*{20}l} {\Gamma_{11} } & {\Gamma_{12} } & {\Gamma_{13} } & {\Gamma_{14} } & {\Gamma_{15} } \\ {\Gamma_{21} } & {\Gamma_{22} } & {\Gamma_{23} } & {\Gamma_{24} } & {\Gamma_{25} } \\ \vdots & \vdots & \vdots & \vdots & \vdots \\ {\Gamma_{101} } & {\Gamma_{102} } & {\Gamma_{103} } & {\Gamma_{104} } & {\Gamma_{105} } \\ \end{array} } \right],$$

where *Γ*_11_ − *Γ*_55_ are associated with the condition at *x* = 0 and *Γ*_61_ − *Γ*_105_ are associated with the condition at *x* = *L*.

For a truncated conical shell clamped at the small radius (*x* = 0) and simply supported at the large radius (*x* = *L*) which is denoted by “CS” in this paper, *Γ*_11_ − *Γ*_105_ are presented as below:

39 $$\begin{array}{*{20}l} {\text{Clamped}}\,\left( {\text{C}} \right){:} \hfill \\ \Gamma_{11} = \Gamma_{22} = \Gamma_{33} = \Gamma_{44} = \Gamma_{55} = I_{1} , \hfill \\ \Gamma_{12} = \Gamma_{13} = \Gamma_{14} = \Gamma_{15} = \Gamma_{21} = \Gamma_{23} = \Gamma_{24} = \Gamma_{25} = \Gamma_{31} = \Gamma_{32} = \Gamma_{34} \hfill \\ = \Gamma_{35} = \Gamma_{41} = \Gamma_{42} = \Gamma_{43} = \Gamma_{45} = \Gamma_{51} = \Gamma_{52} = \Gamma_{53} = \Gamma_{54} = \left\{ 0 \right\}_{1 \times N} , \hfill \\ {\text{Simply}}\,{\text{supported}}\,\left( {\text{S}} \right){:} \hfill \\ \begin{array}{*{20}l} {\Gamma_{61} = A_{11} A_{N} + \frac{{A_{12} \sin \alpha }}{b}I_{N} ,} & {\Gamma_{94} = D_{11} A_{N} + \frac{{D_{12} \sin \alpha }}{b}I_{N} ,} & {\Gamma_{72} = \Gamma_{83} = \Gamma_{105} = I_{N} ,} \\ \end{array} \hfill \\ \Gamma_{62} = \Gamma_{63} = \Gamma_{64} = \Gamma_{65} = \Gamma_{71} = \Gamma_{73} = \Gamma_{74} = \Gamma_{75} = \Gamma_{81} = \Gamma_{82} = \Gamma_{84} \hfill \\ = \Gamma_{85} = \Gamma_{91} = \Gamma_{92} = \Gamma_{93} = \Gamma_{95} = \Gamma_{101} = \Gamma_{102} = \Gamma_{103} = \Gamma_{104} = \left\{ 0 \right\}_{1 \times N} , \hfill \\ \end{array}$$

where subscripts 1 and *N* respectively stand for the first and last rows of each matrix.

Simultaneous solutions of Eqs. (34) and (37) generate an inequality between the numbers of the equations and unknown variables (non-square matrices in the final eigenvalue equation)^38^. To remove this inequality, let us divide the grid points into two sets: the boundary points (*x*_1_ and *x*_N_) and the domain ones (*x*_2_, *x*_3_,…, *x*_*N*−2_, *x*_*N*−1_). Ignoring the satisfaction of governing equations at the boundary points, Eq. (34) can be represented as below:

40 $$\left[ {\tilde{K}} \right]\left\{ c \right\} = \omega^{2} \left[ {\tilde{M}} \right]\left\{ c \right\},$$

in which the sign ~ is utilized to show the created non-square matrices.

Obviously, ignoring the satisfaction of governing equations at the boundary points decreases the accuracy of the solution. But, in Gauss–Lobatto–Chebyshev distribution pattern (Eq. (32)), there is an agglomeration of the points at two ends of the domain which contains the boundary points. Consequently, the side effect of the above-mentioned assumption dramatically decreases^38,41^.

By partitioning the matrices to separate the columns associated with the boundary and domain points, Eqs. (37) and (40) can be represented as below:

41 $$\left[ {\tilde{K}} \right]_{d} \left\{ c \right\}_{d} + \left[ {\tilde{K}} \right]_{b} \left\{ c \right\}_{b} = \omega^{2} \left( {\left[ {\tilde{M}} \right]_{d} \left\{ c \right\}_{d} + \left[ {\tilde{M}} \right]_{b} \left\{ c \right\}_{b} } \right),$$

42 $$\left[ \Gamma \right]_{b} \left\{ c \right\}_{b} + \left[ \Gamma \right]_{d} \left\{ c \right\}_{d} = \left\{ 0 \right\},$$

where subscripts ''*b*'' and “*d*” respectively show the boundary and domain points. By substituting Eq. (42) into Eq. (41), the following eigenvalue equation can be obtained:

43 $$\left[ {K^{*} } \right]\left\{ c \right\}_{d} = \omega^{2} \left[ {M^{*} } \right]\left\{ c \right\}_{d} ,$$

in which

44 $$\begin{array}{*{20}l} {\left[ {K^{*} } \right] = \left[ {\tilde{K}} \right]_{b} \left[ R \right] + \left[ {\tilde{K}} \right]_{d} ,} & {\left[ {M^{*} } \right] = \left[ {\tilde{M}} \right]_{b} \left[ R \right] + \left[ {\tilde{M}} \right]_{d} ,} & {\left[ R \right] = - \left[ \Gamma \right]_{b}^{ - 1} \left[ \Gamma \right]_{d} .} \\ \end{array}$$

Solving Eq. (43) results the shell’s natural frequencies (*ω*). The natural frequencies in various vibrational modes are denoted by *ω*_nm_ in which the first subscript (*n*) is the circumferential wave number (Eq. (27)), and the second one (*m*) is employed to indicate the meridional mode number. Also, the following definition is utilized in this paper to present the natural frequencies in a dimensionless form:

45 $$\lambda_{nm} = \omega_{nm} a\sqrt {\frac{{\rho_{f} }}{{E_{f} }}} ,$$

where *ρ*_f_ and *E*_f_ sequentially stand for the density and elastic modulus of the face sheets.

## Numerical results

Numerical results are provided in this part of the paper for the presented solution. In what follows, except as expressly stated, a CS conical shell is considered with the geometrical characteristics *a* = 0.5 m, *α* = 45°, *h*/*a* = 0.1, *L*/*a* = 4, and *h*_c_/*h* = 0.5. The shell consists of an FGP core of distribution pattern SI, *e*_1_ = 0.5, and *B*_0_ = 0.5. The mechanical properties of the FGP core are *ρ*_0_ = 2700 kg/m^3^, *ν* = 0.25, *E*_0_ = 60 GPa, and *α*_0_ = 0.19^16,42^ and those of the face sheets are *ρ*_*f*_ =2707 kg/m^3^, *ν*_*f*_ =0.3, and *E*_*f*_ =70 GPa.

### Convergence analysis and validation

**Figure 3:**
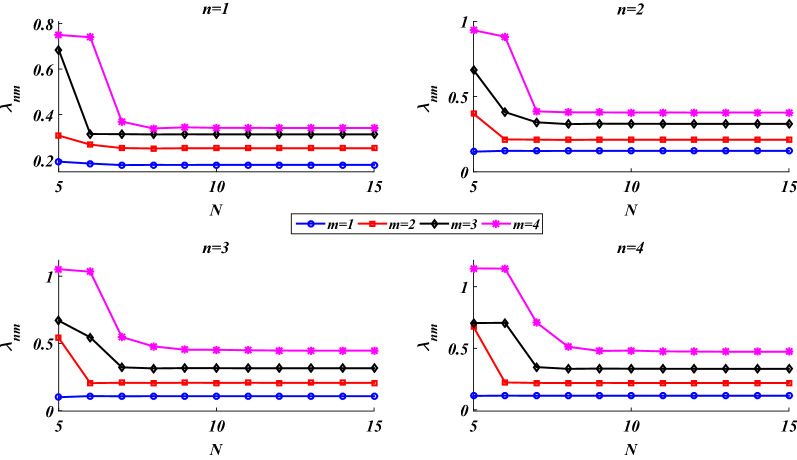
Convergence analysis of the presented solution.

The convergence analysis of the presented solution is examined in Fig. 3 for some vibrational modes. This figure shows that as the number of grid points grows (*N* in Eqs. (30)–(32)), values of the natural frequencies converge rapidly which approves the convergence analysis of the numerical solution performed in the meridional direction. In what follows, numerical examples are presented for *N* = 11.

In order to check the precision of the presented solution, two examples are provided in this section. As the first example, consider an isotropic homogenous (*ν* = 0.3) truncated conical shell of *α* = 45°, *L*sin*α*/*b* = 0.5, and *h*/*b* = 0.01. For *m* = 1 and various values of the circumferential wave number, dimensionless natural frequencies (*Ω*_nm_ = *ω*_nm_*b*[*ρ*(1 − *ν*^2^)/*E*]^0.5^) are tabulated in Table 2 versus those reported by Liew et al.^43^. As this table reveals, the results are in high matching which confirms the precision of the presented solution.

**Table 2:** First dimensionless natural frequency (Ω_n1_ = *ω*_n1_*b*[*ρ*(1 − *ν*^2^)/*E*]^0.5^) of a homogenous isotropic truncated conical shell with various boundary conditions (*ν* = 0.3, *α* = 45°, *L*sin*α*/*b* = 0.5, *h*/*b* = 0.01).

		*n* = 1	*n* = 2	*n* = 3	*n* = 4	*n* = 5	*n* = 6	*n* = 7	*n* = 8	*n* = 9
CC	Present	0.8117	0.6694	0.5426	0.4563	0.4085	0.3956	0.4133	0.4555	0.5159
	Liew et al.^43^	0.8120	0.6696	0.5428	0.4565	0.4088	0.3961	0.4141	0.4567	0.5175
SC	Present	0.8115	0.6611	0.5246	0.4318	0.3825	0.3733	0.3979	0.4467	0.5115
	Liew et al.^43^	0.8113	0.6610	0.5244	0.4316	0.3832	0.3732	0.3980	0.4472	0.5124
CS	Present	0.7094	0.6473	0.5200	0.4159	0.3589	0.3445	0.3641	0.4082	0.4692
	Liew et al.^43^	0.7095	0.6473	0.5199	0.4158	0.3589	0.3446	0.3644	0.4088	0.4701
SS	Present	0.5460	0.6308	0.5062	0.3942	0.3339	0.3235	0.3507	0.4014	0.4661
	Liew et al.^43^	0.5462	0.6309	0.5061	0.3941	0.3337	0.3235	0.3510	0.4019	0.4671

As the second example, consider an SS isotropic homogenous conical shell (*ν* = 0.3) of *L*sin*α*/*b* = 0.25 and *h*/*b* = 0.01. For two selected values of the semi-vertex angle and various values of the circumferential wave number (*n* = 1,2,..,9), the dimensionless natural frequencies of the shell (Ω_nm_ = *ω*_nm_*b*[*ρ*(1 − *ν*^2^)/*E*]^0.5^) are presented for *m* = 1 in Table 3 against those reported by Dai et al.^44^. This table confirms that results are in high matching which confirms the precision of the presented numerical solution.

**Table 3:** First dimensionless natural frequency (Ω_n1_ = *ω*_n1_*b*[*ρ*(1 − *ν*^2^)/*E*]^0.5^) of an SS isotropic homogenous truncated conical shell with various values of the semi-vertex angle (*ν* = 0.3, *L*sin*α*/*b* = 0.25, *h*/*b* = 0.01).

		n = 1	n = 2	n = 3	n = 4	n = 5	n = 6	n = 7	n = 8	n = 9
α = 30°	Present	0.5922	0.7908	0.7281	0.6348	0.5524	0.4940	0.4640	0.4628	0.4871
	Dai et al.^44^	0.5922	0.7909	0.7282	0.6349	0.5525	0.4941	0.4641	0.4633	0.4879
α = 60°	Present	0.4748	0.5715	0.5994	0.6044	0.6066	0.6144	0.6325	0.6627	0.7056
	Dai et al.^44^	0.4754	0.5721	0.6001	0.6053	0.6075	0.6156	0.6340	0.6646	0.7080

### Parametric study

**Figure 4:**
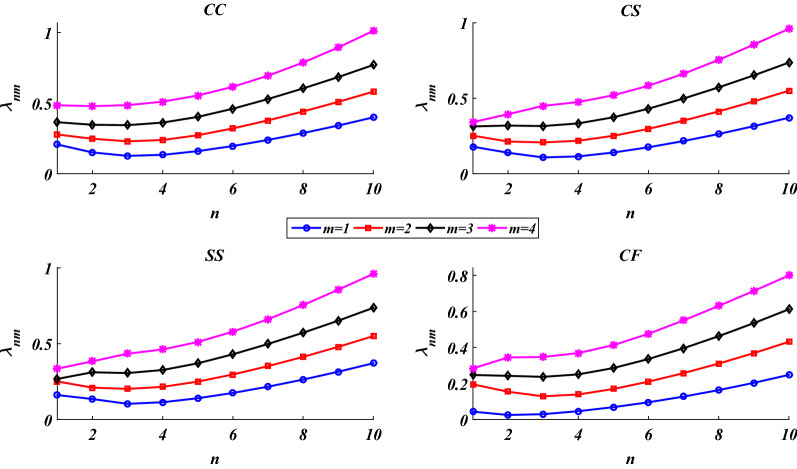
Dependency of the natural frequencies on the circumferential wave number.

The dependency of the shell’s natural frequencies on the circumferential wave number is examined in Fig. 4. As this figure reveals, by increasing the circumferential wave number, the natural frequencies experience an initial reduction followed by increasing growth. In other words, there is a special value of the circumferential wave number which provides the lowest natural frequency (the fundamental frequency, *λ*_n1_). As the circumferential wave number increase, the shell experiences various shapes of harmonic functions (sinus or cosinus) in the circumferential direction. Depending on the geometrical parameters of the shell, boundary conditions, and the meridional mode number (*m*), there is a specific shape of the shell in the circumferential associated with a specific value of the circumferential wave number which provides the minimum rigidity and consequently the minimum natural frequency.

Table 4 shows the influences of the boundary conditions on the shell’s natural frequencies. As observed, the highest natural frequency at each vibrational mode belongs to the CC shell which means that the more constrained boundaries result in higher natural frequencies. It can be explained by the higher rigidity of the shell under CC boundary conditions. A comparison between the CS, SC, and FC shells reveals that the natural frequencies of the SC shell are greater than the corresponding ones of the CS shell and in some vibrational modes, the natural frequency of the FC shell is greater than the natural frequency of the CS one. It means that the boundary condition at *x* = *L* (the shell’s large radius) has a stronger effect on the natural frequencies of the conical shells rather than the boundary condition at *x* = 0 (the shell’s small radius). It can be explained by the higher perimeter of the shell’s edge at its large radius in comparison with the small one.

**Table 4:** Dependency of the natural frequencies on the boundary conditions.

	*n* = 1				*n* = 2			
	CC	CS	SC	FC	CC	CS	SC	FC
*m* = 1	**0.2068**	0.1788	0.2050	0.1955	**0.1505**	0.1388	0.1461	0.1218
*m* = 2	**0.2777**	0.2519	0.2726	0.2661	**0.2475**	0.2130	0.2421	0.2047
*m* = 3	**0.3649**	0.3131	0.3556	0.3338	**0.3466**	0.3189	0.3370	0.2886
*m* = 4	**0.4849**	0.3417	0.4627	0.4055	**0.4788**	0.3943	0.4612	0.3914

	*n* = 3				*n* = 4			
	CC	CS	SC	FC	CC	CS	SC	FC
*m* = 1	**0.1263**	0.1076	0.1222	0.1145	**0.1335**	0.1146	0.1322	0.1316
*m* = 2	**0.2294**	0.2070	0.2226	0.1944	**0.2387**	0.2183	0.2345	0.2281
*m* = 3	**0.3439**	0.3155	0.3331	0.2875	**0.3622**	0.3333	0.3537	0.3314
*m* = 4	**0.4857**	0.4491	0.4687	0.3983	**0.5104**	0.4747	0.4965	0.4439

Significance values are given in bold.

Table 5 is presented to investigate the effect of the pore distribution pattern on the shell’s natural frequencies. As this table shows, in most vibrational modes, the highest natural frequency belongs to the SI pattern. As depicted in Fig. 2, in the SI pattern the big pores are distributed close to the shell’s neutral surface (SI) which leads to the minimum reduction in the shell’s flexural rigidity. It is noteworthy that alongside the flexural rigidity, the rotational inertia (*I*_2_ in Eq. (17)) can be affected by the pore distribution pattern. Consequently, in some cases, the highest natural frequency belongs to the SII pattern which has the minimum rotational inertia.

**Table 5:** Dependency of the natural frequencies on the pore distribution pattern.

	*n* = 1			*n* = 2		
	UD	SI	SII	UD	SI	SII
*m* = 1	0.1781	0.1788	**0.1796**	0.1382	0.1388	**0.1393**
*m* = 2	0.2509	0.2519	**0.2524**	0.2121	**0.2130**	**0.2130**
*m* = 3	0.3120	0.3131	**0.3149**	0.3171	**0.3189**	0.3173
*m* = 4	0.3399	**0.3417**	0.3408	0.3928	0.3943	**0.3960**

	*n* = 3			*n* = 4		
	UD	SI	SII	UD	SI	SII
*m* = 1	0.1070	**0.1076**	0.1072	0.1138	**0.1146**	0.1134
*m* = 2	0.2059	**0.2070**	0.2061	0.2169	**0.2183**	0.2161
*m* = 3	0.3135	**0.3155**	0.3128	0.3310	**0.3333**	0.3293
*m* = 4	0.4460	**0.4491**	0.4440	0.4713	**0.4747**	0.4683

Significance values are given in bold.

**Figure 5:**
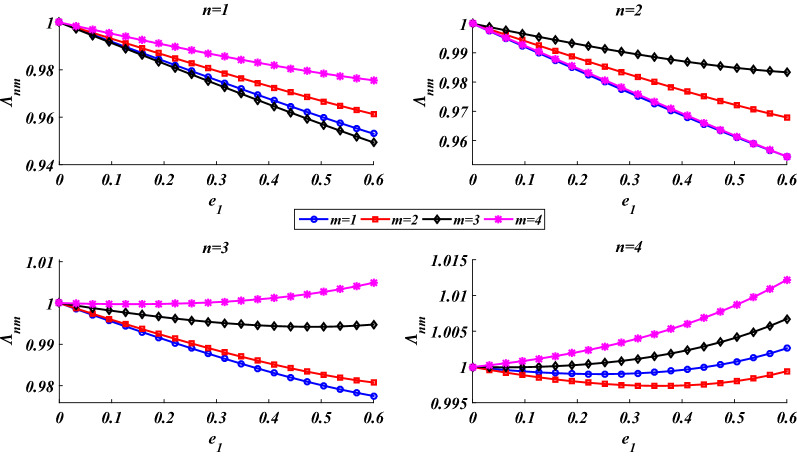
Dependency of the natural frequencies on the porosity parameter.

The dependency of the shell’s natural frequencies on the porosity parameter is examined in Fig. 5. By growing the porosity parameter, the size of the pore increases which decreases both the rigidity and inertia of the shell. Consequently, as the porosity parameter grows, depending on the vibrational mode, either increase or decrease in the natural frequency can be seen. As shown in this figure, due to the confrontation between the reductions in the rigidity and inertia of the shell, an increase in the porosity parameter has no remarkable effect on the natural frequencies. Thus, to make it possible to show the small variations of the natural frequencies in different vibrational modes, simultaneously, the following frequency parameter is defined:

46 $$\Lambda_{nm} = \frac{{\lambda_{nm} }}{{\left. {\lambda_{nm} } \right|_{{e_{1} = 0}} }}.$$

Figure 5 confirms that for (*n,m*) = 1, 2, 3, 4, by increasing the porosity parameter from zero to *e*_1_ = 0.6, the maximum reduction and increase in the natural frequencies are less than 5% and 1.5%, respectively.

**Figure 6:**
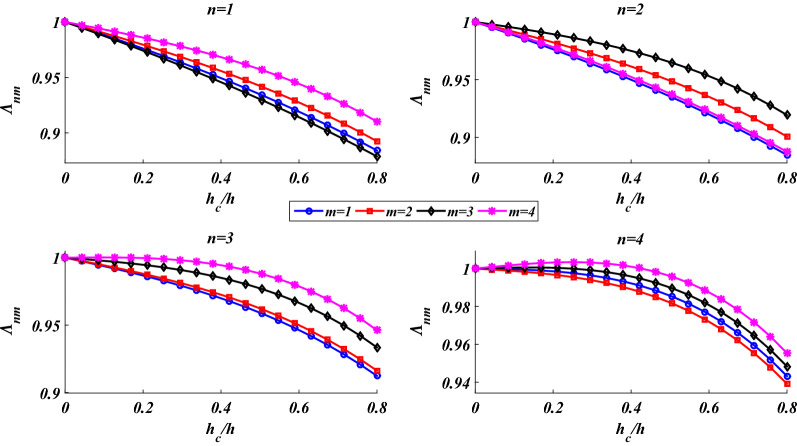
Dependency of the natural frequencies on the thickness of the FGP core.

For a specified value of the shell’s thickness, Fig. 6 shows the effect of the thickness of the FGP core on the shell’s natural frequencies. By increasing the thickness of the FGP core, both inertia and rigidity of the shell decrease. Thus, as the thickness of the FGP core grows, depending on the vibrational mode, either increase or decrease in the natural frequency can be seen.

As this figure shows, due to the confrontation between the reductions in the inertia and rigidity of the shell, an increase in the shell’s thickness has no considerable influence on the natural frequencies. Consequently, to make it possible to show the small variations of the natural frequencies in various vibrational modes, simultaneously, the frequency parameter is defined as below:

47 $$\Lambda_{nm} = \frac{{\lambda_{nm} }}{{\left. {\lambda_{nm} } \right|_{{h_{c} = 0}} }}.$$

Figure 6 shows that for (*n,m*) = 1,2,3,4, by increasing the thickness of the FGP core from zero to 0.8* h*, the maximum reduction in the natural frequencies is less than 12%.

**Figure 7:**
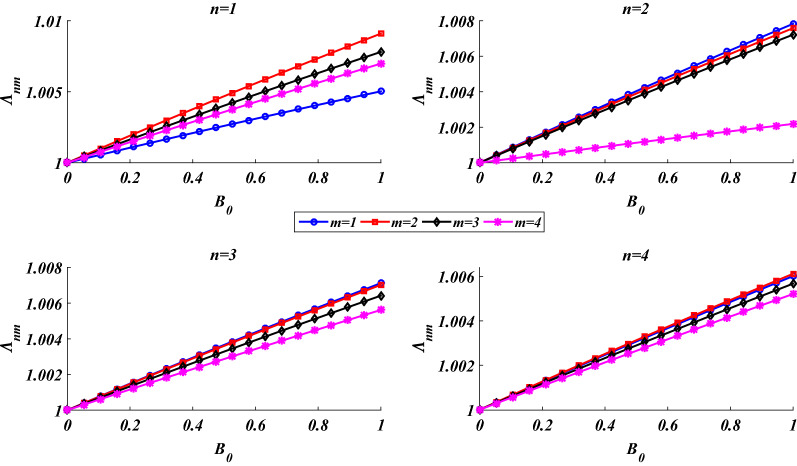
Dependency of the natural frequencies on the compressibility of the pore fluid.

Figure 7 is provided to examine the effect of the compressibility of the pore fluid on the shell’s natural frequencies. As observed, by increasing the Skempton parameter (decreasing the compressibility of the pore fluid), a small growth occurs in the natural frequencies which can be explained by the small growth in the rigidity of the shell. As this figure shows, for (*n*,*m*) = 1, 2, 3, 4, by increasing the Skempton parameter from the minimum possible value (*B*_0_ = 0) to the maximum possible value (*B*_0_ = 1), the maximum increase in the natural frequencies is less than 1%.

It should be noticed that due to the weak effect of the compressibility of the pore fluid on the natural frequencies, to make it possible to show the small variations of the natural frequencies in different vibrational modes, simultaneously, the frequency parameter is defined as follows:

48 $$\Lambda_{nm} = \frac{{\lambda_{nm} }}{{\left. {\lambda_{nm} } \right|_{{B_{0} = 0}} }}.$$

## Conclusions

The free vibrational analysis of a sandwich truncated conical shell with a saturated FGP core and two same homogenous isotropic face sheets was examined. The mechanical behavior of the saturated FGP core and mathematical modeling of the shell was performed based on Biot’s theory and the FSDT respectively. Three different distribution patterns of the pores were investigated including a uniform distribution pattern and two non-homogenous symmetric ones. The main findings of the paper can be listed as below:

- The more constrained boundaries at the ends of the shell result in higher natural frequencies.
- The boundary condition at the shell’s large radius has a stronger effect on the natural frequencies of the conical shells rather than the boundary condition at the shell’s small radius.
- When the bigger pores are located close to the neutral surface of the shell, the natural frequencies become greater.
- By increasing the porosity parameter and thickness of the FGP core, either growth or reduction in the natural frequency can be seen. It depends on the vibrational mode.
- The lower compressibility of the pore fluid results in higher natural frequencies. But, the maximum increase is less than 1%.
